# An Unusual Case of Hyperpigmented Maculopapular Rash with Unmasking of Lepromatous Leprosy after Steroid Cessation

**DOI:** 10.4269/ajtmh.20-0510

**Published:** 2020-10

**Authors:** Shafeeqa Hassan, Balram Rathish, Faiz Mukthar

**Affiliations:** 1 Leprology and Venereology, Department of Dermatology, Lourdes Hospital, Ernakulam, India;; 2 Department of Internal Medicine, Aster Medcity, Kochi, India

**Figure 1.**
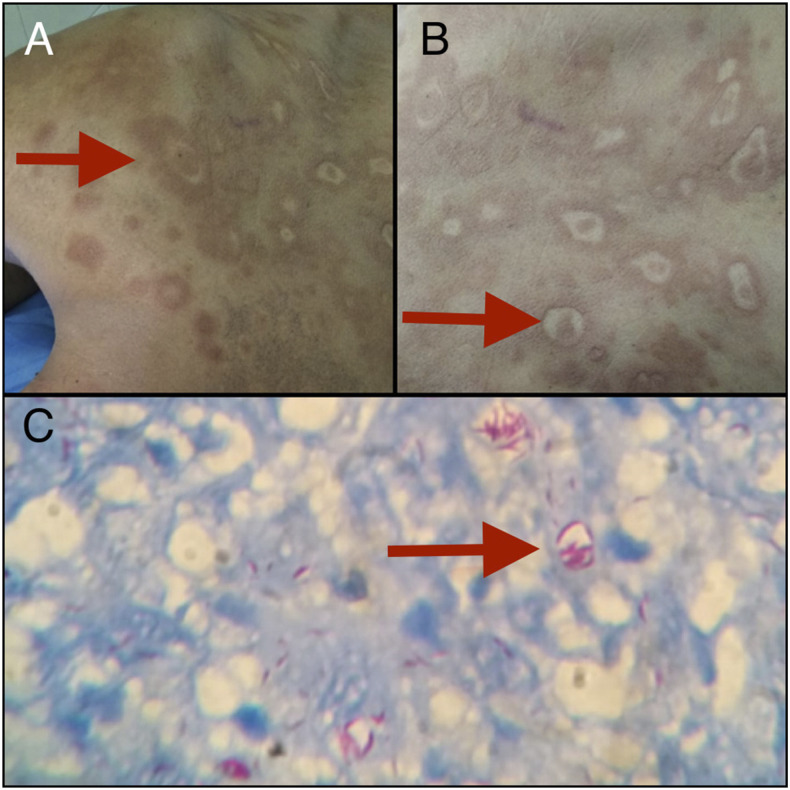
(**A**) Multiple large, annular, hypopigmented, atrophic macules and plaques with well-defined, erythematous, raised borders. (**B**) “Inverted saucer”–shaped punched-out lesions. (**C**) Modified Ziehl–Neelsen staining showing acid-fast rod-shaped organism and globi. This figure appears in color at www.ajtmh.org.

A 70-year-old man with no comorbidities presented with a 1-year history of rash on his back. He was prescribed mometasone and tacrolimus ointment from elsewhere for suspected vitiligo with no respite. Examination showed hyperpigmented maculopapular rashes on the upper back. After stopping all medications, the rash evolved into multiple large, annular, hypopigmented, atrophic macules and plaques with well-defined, erythematous, raised borders (Figure 1A). There were also “inverted saucer”–shaped punched-out lesions (Figure 1B). Touch and temperature sensations were intact over the lesions, but these were impaired in a glove-and-stocking pattern. Nerve conduction studies showed impaired nerve conduction velocities, amplitude, and latencies in predominantly sensory nerves. There were no palpable nerves. Skin biopsy was taken. Modified Ziehl–Neelsen staining showed acid-fast rod-shaped organisms with parallel sides and rounded ends, and globi, consisting of clumps of bacilli in capsular material (Figure 1C). He was diagnosed with lepromatous leprosy. Treatment was started with rifampicin, clofazimine, and dapsone following which he showed clinical improvement. Initial misdiagnosis and treatment with steroid further confounded the diagnosis of leprosy.

